# The clinical role of positive latex IgE in patients with food/pollen adverse reactions

**DOI:** 10.1186/2045-7022-1-S1-P82

**Published:** 2011-08-12

**Authors:** Arianna Aruanno, Eleonora Nucera, Alessandro Buonomo, Tiziana De Pasquale, Valentina Pecora, Sonia Musumeci, Amira Colagiovanni, Vito Sabato, Lucilla Pascolini, Anna Giula Ricci, Angela Rizzi, Domenico Schiavino

**Affiliations:** 1 Policlinico A Gemelli, Allergy Department, Rome, Italy

## 

Natural rubber latex allergy (NRL-A) is an international problem of public health. About 50-60% of NRL-A patients may present adverse reactions after ingestion cross-reacting vegetable foods. This condition is called "Latex-fruit Syndrome" and is matter of research. The aim of our study is distinguishing between clinical/subclinical latex-fruit syndrome and cross-sensitization to latex and food/pollens allergens on the basis of latex recombinant allergens. We studied 19 patients with food hypersensitivity and serological evidences of NRL sensitization. They underwent an accurate allergological evaluation (skin prick test with latex, food and pollen extracts, specific IgE to latex and recombinant allergens, challenge provocation tests). The patients were divided in two groups: group 1) 9 patients allergic to fruits/vegetables and/or pollens, with serological, but not clinical NRL-A; group 2) 10 patients with clinical and serological latex and fruits/vegetables allergy. The same number of patients was positive to pollens in each group. All group 1 patients presented negative provocation challenges and a monosensitization to Hev b8 (and other recombinant profilins), probably linked to a cross-sensitization to pollens and foods. These data suggest that profilin sensitization determines false positivity from NRL-A diagnostic test. Consequently the observed in vitro reactivity to NRL is not clinically relevant in patients with adverse food reactions and it does not determine always a latex-fruit syndrome. Instead, in group 2 we observed positive NRL provocation challenges and sensitization to rHev b 8 , but also to other recombinant latex allergens (rHev b 5, rHev b 6.01, rHev b 6.02). These patients probably presented a latex-fruit syndrome.

We supposed that the available panel of recombinant latex allergens could be effective to demonstrate a significant difference between latex-fruit syndrome and cross-sensitization to latex and food/pollens allergens. So further investigations are needed with a larger group of patients.

